# Progressive Loss of Muscle Strength: The Effects of Ageing and Sarcopenia on Muscle Function in Older Females

**DOI:** 10.3390/jcm14207276

**Published:** 2025-10-15

**Authors:** Katarzyna Nowakowska-Lipiec, Hanna Zadoń, Robert Michnik, Agnieszka Nawrat-Szołtysik

**Affiliations:** 1 Department of Biomechatronics, Faculty of Biomedical Engineering, Silesian University of Technology, 41-800 Zabrze, Poland; hanna.zadon@polsl.pl (H.Z.); robert.michnik@polsl.pl (R.M.); 2 Department of Clinical Engineering, Academy of Silesia, 40-555 Katowice, Poland; 3 Institute of Physiotherapy and Health Sciences, Academy of Physical Education, 40-065 Katowice, Poland; a.nawrat-szoltysik@awf.katowice.pl; 4 Department of Physiotherapy in Internal Medicine, Faculty of Physiotherapy, Academy of Physical Education, 40-065 Katowice, Poland

**Keywords:** ageing, sarcopenia, muscle strength, Anybody Modeling System, numerical simulations

## Abstract

**Background/Objectives**: Sarcopenia is the progressive loss of muscle mass, strength, and/or function, leading to reduced physical performance, independence, and social participation. This study aimed to analyze the effects of age-related muscle strength loss and sarcopenia on muscle function during standing in older females. **Methods**: This study included experimental and modeling analyses using the AnyBody Modeling System in 20 older females. Based on DEXA results, participants were divided into older females without sarcopenia (OF) and with sarcopenia (OFS). Body posture while standing was assessed using the Zebris APGMS Pointer system. A model of muscular strength changes due to natural aging and progressive sarcopenia was developed based on literature data. The experimental results informed model studies in the AnyBody Modeling System, which incorporated changes in body posture and loss of muscle strength. **Results**: Total muscle activity during standing increases with age; however, this increase is significantly more pronounced in individuals with sarcopenia, especially after the age of 65. At 65, total muscle activity was 15% higher in the OFS model than in the OF model, while the difference was 44% at 80. After age 65, muscle fatigue increased considerably with progressive sarcopenia. At age 80, muscle fatigue while standing with sarcopenia can be more than three times higher than in those without sarcopenia. **Conclusions**: Aging leads to increased muscle activity while standing, and sarcopenia further amplifies this effect, particularly in individuals over 65. Modeling results highlight the pronounced impact of sarcopenia on muscle fatigue, demonstrating its significant functional consequences in older females.

## 1. Introduction

Current demographic projections [1] show a steady increase in the proportion of older people (65+) within populations of most societies, with numbers expected to exceed 1 billion by 2030 and 1.6 billion by 2050, comprising almost 2.5 billion older people globally by the end of the 21st century [1]. One of the principal challenges facing modern medicine is the global prevalence of lifestyle diseases often associated with this growth.

Aging is an exceptionally complex, irreversible, dynamic, and widespread phenomenon whose complexity depends on the intricacies of the human body. Physical fitness affects an individual’s ability to perform daily activities and overall independence and is a critical element in quality-of-life maintenance in old age. As such, preserving fitness is highly desirable as it reduces the need for reliance on others for medical and long-term care [2,3]. However, with age, many people experience a gradual decline in muscle mass and strength, leading to reduced physical activity and motor function deterioration [4,5]. One of the primary causes of such changes is sarcopenia.

Sarcopenia involves a concurrent widespread and progressive degeneration in skeletal muscle mass and strength and/or function, resulting in a decline in overall physical performance [6,7], leading to loss of independence and reduced social activity. Those with sarcopenia are at increased risk of falls [8,9,10] and mortality [6,8,9,11,12] and are more likely to require hospitalization and stays in long-term care facilities, placing a heavy burden on their families and the health care budget [3]. The growing global elderly population makes sarcopenia an increasingly serious threat, affecting the quality of life and independence of older people, as sarcopenia is most commonly diagnosed in older people. However, it can also occur in other diseases that are not age-related [6]. In 2016, the International Statistical Classification of Diseases and Related Health Problems, 10th revision (ICD-10), recognized sarcopenia as a disease entity under code M62.84 [13].

Prevalence statistics vary by diagnostic method, age group, and region, but overall data suggest that sarcopenia affects a significantly higher proportion of older people (10% to 16% worldwide), which increases with age to impact up to 50% of those aged over 80 [14]. However, people with underlying conditions experience sarcopenia, ranging from 18% of diabetes patients to 66% of those suffering from inoperable esophageal cancer [15]. Studies show that sarcopenia is more common in females than males, particularly after menopause, when hormonal changes affect muscle metabolism. Females are also more prone to osteoporosis, which contributes to muscle loss [16].

In humans, peak muscle mass and strength occur in early adulthood, usually between the ages of 20 and 30. Subsequently, muscle mass and strength are progressively lost until the age of 50, and the rate of decline then accelerates [17]. Skeletal muscle mass and strength reduce in those with sarcopenia from the fourth decade, with up to 50% of mass lost by the eighth decade. Muscle strength is estimated to reduce by 12% to 15% per decade after age 50 [5], with muscle strength declining more rapidly in subsequent decades [5,17,18,19,20]. This decline in muscle strength leads to decreased functional status and physical performance. Sarcopenia is considered a component of frailty syndrome [4,21], with muscle weakness, especially in the postural muscles, causing impaired balance, an increased risk of falls, and difficulties performing activities of daily living [8,9,10]. Such reduced mobility in older people increases the risk of disability, fractures, and premature death [9,12].

Recent years have seen increasing interest in research assessing the functional status and physical performance of people with sarcopenia [22], with such studies also focusing on identifying risk factors, tailoring appropriate therapeutic programs, and the effectiveness of interventions that can improve quality of life [8,10,23,24]. With advancing age, muscles lose mass and strength, which limits their functional capacity and recovery potential. These changes negatively affect daily activities and overall quality of life [6,7]. Assessment of these changes, especially in people with progressive sarcopenia, is crucial. Knowledge of musculoskeletal loading and muscle functioning in older people is the basis for effective prevention, diagnosis and treatment, contributing to improved health and quality of life. Despite the growing interest in sarcopenia research, there remains a lack of detailed studies on musculoskeletal load modeling and evaluation in people with the disease, with only one paper addressing the topic [25]. This study used modeling to determine the effects of muscle aging and sarcopenia on muscle recruitment patterns and spinal loads and found that compressive loads in the upper thoracic spine and shear loads throughout the spine significantly increased during the severe and very severe disease stages. Furthermore, activity increased in almost all of the muscles in the main trunk muscle group in sarcopenic models, reaching 100% of the available strength [25]. Considering the increasing number of older people in society, there is a clear need for further research in this area.

The aim of this study was to analyze the effects of age-related muscle strength loss and sarcopenia on muscle function while standing in older females.

We hypothesize that aging is associated with increased muscle activity during standing and that sarcopenia leads to a dynamic increase in this activity, particularly after age 65. We also hypothesize that, after age 65, there is a marked increase in muscle fatigue while maintaining standing in people with progressive sarcopenia.

## 2. Materials and Methods

**Figure 1:**
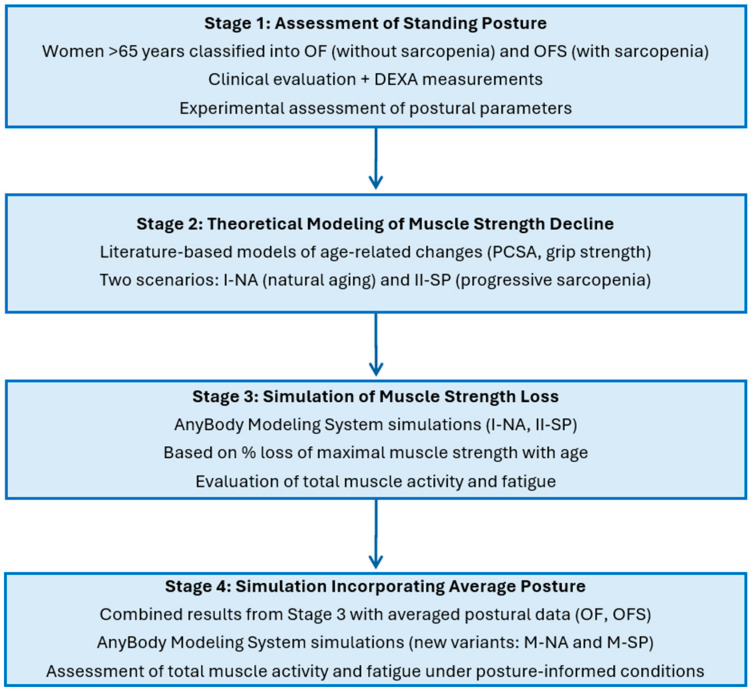
Four-stage research process: assessment of standing posture, theoretical modeling of muscle strength decline due to natural aging and progressive sarcopenia; musculoskeletal simulation of age-related muscle strength loss; and simulations incorporating body posture. Legend: OF—older females without sarcopenia; OFS—older females with sarcopenia; PCSA—physiological cross-sectional area of the muscle; I-NA—simulation variant accounting for the aging process defined by a model that assumes a percentage loss of maximum muscular strength with age due to the natural aging process; II-SP—simulation variant accounting for the aging process defined by a model assuming a percentage loss of maximal muscle strength with age in individuals with progressive sarcopenia; M-NA—model of the posture of OF and strength loss associated with natural aging processes; M-SP—model the posture of OFS and strength loss associated with progressive sarcopenia; Total muscle activity—the dimensionless sum of the activity of all muscle actions building the model; Total muscle fatigue—a dimensionless indicator from the optimization function, where higher values reflect greater fatigue.

The research procedure included four main stages (Figure 1):

- Assessment of standing posture—Women aged over 65 were classified into groups with sarcopenia (OFS) and without sarcopenia (OF) based on clinical evaluation and DEXA measurements. Postural parameters were then assessed experimentally.
- Theoretical modeling of muscle strength decline—Models were developed to represent progressive muscle strength loss across consecutive decades of life, based on literature data describing age-related changes in physiological cross-sectional area (PCSA) and grip strength. Two strength-decline scenarios were considered: natural aging (Variant I-NA) and progressive sarcopenia (Variant II-SP).
- Simulation of age-related muscle strength loss—Using the AnyBody Modeling System, simulations were conducted for both strength-decline variants (I-NA and II-SP) across consecutive decades to evaluate changes in total muscle activity and muscle fatigue, based on the percentage loss of maximal muscle strength with age.
- Simulation incorporating average body posture—Results from Stage 3 were combined with averaged experimental postural data from the OF (older females without sarcopenia) and OFS (older females with sarcopenia) groups to generate posture-specific simulation variants (M-NA and M-SP). This allowed for the assessment of muscle activity and fatigue under realistic, posture-informed conditions throughout aging.

### 2.1. Materials

The study included 20 older females aged 69 to 88 years (78 ± 7 years, 1.57 ± 0.06 m, 67.4 ± 12.3 kg, body mass index [BMI]: 27.31 ± 5.02 kg/m^2^). Based on the dual-energy X-ray absorptiometry (DEXA) results, participants were divided into two groups: older females without sarcopenia (OF, *n* = 10) and older females with sarcopenia (OFS, *n* = 10).

Inclusion criteria were age over 65 years, no injuries in the previous three months, and no posture-related surgery. Informed consent for study inclusion was obtained from all participants. The ethics committee of the Jerzy Kukuczka Academy of Physical Education in Katowice, Poland, approved the study (protocol number 5/2018, dated 26 October 2018).

### 2.2. Experimental Tests

The participants underwent a densitometry (DEXA) scan to measure body composition and muscle mass. Before the test, subjects removed all metal objects that could interfere with the results. During the test, participants lay still on the scanner table in a supine position with legs slightly apart and arms placed next to the body. During the scan, the machine sent a beam of radiation through the patient’s body, and the amount of radiation that passed through the tissues was recorded. The difference in X-ray absorption by the bones and soft tissues was used to determine body composition, providing information on the amount of muscle and fat tissue in the body and individual body parts.

The appendicular lean mass index (ALM), the ratio of the sum of the lean mass of the limb to the square of the height determined by DEXA, was used to diagnose sarcopenia. The recommended ALM cut-offs for the European population were adopted from the approach of Newman et al. [26] at 5.78 kg/m^2^ for females. The validity and reliability of DEXA are well-established [27,28].

**Figure 2:**
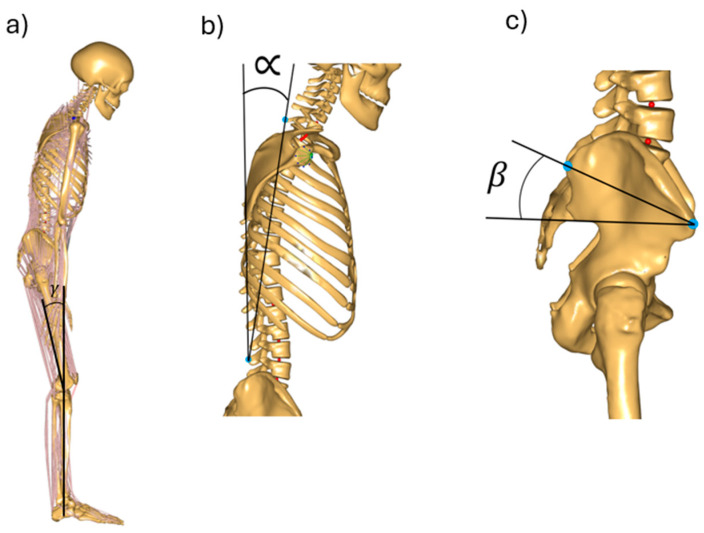
Musculoskeletal model with selected analyzed angles: (**a**) full body model, γ—knee joint flexion angle, (**b**) α—trunk tilted in the sagittal plane, (**c**) β—pelvis aligned in the sagittal plane.

Assessment of trunk and pelvis alignment in the standing position was carried out using the Zebris APGMS Pointer system (Zebris Medical GmbH, Bavaria, Germany). The system consists of an ultrasonic pointer, a measurement sensor with microphones, and a station with input and output sockets. After calibration (pointing to four points forming a 25 cm square with the pointer), the characteristic anatomical points of the subject were identified, i.e., shoulder processes—left and right acromion, within the pelvis, left and right posterior superior iliac spine, left and right anterior superior iliac spine, and left and right iliac crest. A pointer was used to mark the dorsal line of the spine from C7 to L3, and measurements were repeated three times. The angles of trunk tilt in the sagittal plane (α) and pelvic tilt in the sagittal plane (β) were assessed. Angle α was defined as the angle between the straight line defined by the cervical and L3 lumbar vertebrae and the vertical line. Angle β was defined as the angle between the straight line defined by the anterior, superior, and posterior iliac spine and the horizontal straight line (Figure 2). Subjects were also photographed in the sagittal plane, which was used to estimate the flexion angle at the knee joint.

### 2.3. Modeling Studies

The results of the experimental studies were used to perform numerical simulations using a modified model of the whole human body (the so-called standing model) with GRF (Ground Reaction Forces) prediction [29] in the AnyBody Modeling System software (AnyBody Technology, Aalborg, Denmark) version 7.4.

The model of the human musculoskeletal system consisted of bones, represented as non-deformable rigid bodies. They were connected by kinematic pairs of the appropriate class, simulating joints. Muscles were modeled as elastic damping elements. They were attached to body segments to facilitate movement. The spine model included twelve thoracic vertebrae, the thoracic vertebrae and five lumbar vertebrae. These were represented as separate segments connected by intervertebral spherical joints with three degrees of freedom. In addition, the sacrum and pelvis were modeled as rigidly connected elements [30,31]. The lumbar spine model included 188 muscle fascicles representing both abdominal and back muscles. These included the erector spinae (ES), quadratus lumborum, multifidus, transversus abdominis (TrA), rectus abdominis, internal abdominal oblique and external abdominal oblique muscles.

Muscle strength refers to the ability of a muscle to generate force. The strength of each muscle fascicle is calculated as the product of its physiological cross-sectional area (PCSA) and the maximum muscle stress coefficient, which is 90 N/cm^2^ [31,32]. The effect of intra-abdominal pressure is taken into account in the spine model. The intra-abdominal pressure (IAP) model consists of a rigid buckle for attachment to the abdominal muscles and five rigid artificial discs which form the structure for the transverse muscles responsible for the generation of IAP. When the transversus abdominis muscle (TrA) is activated, it controls the anterior–posterior movement and contributes to changes in the volume of the abdominal cavity, which is modeled as a cylinder. The maximum IAP value in the model is limited to 26.6 kPa [33,34]. The IAP muscle is involved in muscle recruitment. When activated, it applies transverse forces to the intervertebral joints. It also contributes to antero-posterior shear forces in these joints. However, it does not produce compressive forces [33,34,35].

Using a traditional inverse dynamics approach combined with static optimization, the researchers calculated the joint reaction forces and the muscle forces for a given position or movement, while minimizing the activation of the muscle recruitment. The standard polynomial optimization criterion [30,32] was used in these simulations. The musculoskeletal system and IAP models used in the study have been validated for the assessment of lumbar spine loads and muscle activation during different positions and movements under physiological conditions [36,37,38,39,40].

Simulations used a standing model alongside a linear scaling method, i.e., the ‘Scaling LengthMass” method (available in the Anybody software repository), which accounts for height, weight, and body fat percentage. Two posture models were developed, one for OF and one for OFS. Simulations were performed for the models under the assumption of constant body weight and height (the mean value for the entire study), while the input data for the simulations included the mean values of the trunk and the pelvic tilt for both groups (Figure 2). The simulations were performed assuming symmetry of the body using AnyMuscleModel, a simple model that assumes a constant value of muscle strength independent of its working conditions. Changes in muscular system strength over the years were considered based on the developed model, which describes the percentage loss of maximum muscle strength in older people due to natural aging processes and progressive sarcopenia.

#### 2.3.1. A Model of Changes in Muscle System Strength Considering the Percentage Loss of Maximal Muscle Strength with Age in Older Females Due to Natural Aging Processes and Progressive Sarcopenia

For the purpose of simulations, a model of changes in musculoskeletal system strength was developed, taking into account the percentage loss of muscle strength with age in older people due to natural aging processes and progressive sarcopenia. The experimental results of Dodds et al. [18] were used to estimate percentage changes. Changes in mean hand grip strength in females were used to estimate percentage changes in musculoskeletal strength over subsequent life years due to natural aging. A model for strength loss in patients with sarcopenia in the later years of life is based on the assumption of higher percentage changes. Data in the literature [5,17,19,20] suggest that skeletal muscle mass and strength decrease in individuals with sarcopenia from the fourth decade, with up to 50% of mass lost in the eighth decade (approximately 15% per decade after age 50) and a more rapid decline in subsequent decades.

Based on data from the literature, the following assumptions were made to model loss of strength in patients with sarcopenia: between 40 and 50 years and 7.5% loss of strength every five years; between 50 and 70 years and 7.5% loss of strength every five years; between 70 and 80 years and 15% loss of strength every five years (resulting in approximately 50% loss of strength at 80 years); between 80 and 90 years and 25% loss of strength every five years. The percentage changes in muscle strength associated with age (natural aging processes) and progressive sarcopenia were determined at five-year intervals from the age of 50 to the age of 90.

#### 2.3.2. Modeling Changes in Musculoskeletal Load Associated with Reduced Muscle Strength Due to Natural Aging and Progressive Sarcopenia in Older Females

Two variants of muscular strength decline in older females were modeled in the Anybody Modeling System:

- Variant I-NA—accounting for the aging process defined by a model that assumes a percentage loss of maximum muscular strength with age due to the natural aging process.
- Variant II-SP—accounting for the aging process defined by a model assuming a percentage loss of maximal muscle strength with age in individuals with progressive sarcopenia.

Age-related changes in the muscular system and progressive sarcopenia were considered in Anybody by introducing specified percentage changes for the strength parameters (SpecificMuscleTensionSpine, StrengthIndexLeg, and SpecificMuscleTensionShoulderArm); that is, measures of the force-generating capacity within the biomechanical model. The percentage decline in muscle strength was applied uniformly to all muscles of the body for each simulated decade of life, reflecting the natural aging process and progressive sarcopenia affecting the entire musculature to the same extent. No age-related changes were modeled, except a reduction in muscle strength. In other words, model parameters such as body weight, height, mass distribution, posture, and passive properties of the joints were the same for all simulation conditions. Simulations were performed for a 50th percentile female model (65 kg and 1.65 m).

The effects of aging and progressive sarcopenia on muscle function were analyzed using the following parameters: total activity of all muscles, muscle activity of selected postural muscles responsible for maintaining stable posture (muscles with the highest activity), and muscle fatigue.

Muscle activity (Activity) is the ratio of muscle force to the maximum force that can be generated by a given muscle/muscle action (strength). The correct muscle activity value for each muscle action in the model ranges from 0 to 1 (dimensionless indicator), with 0 indicating that the muscle action is inactive and a value of 1 corresponding to its maximum activation. Obtaining a muscle activity value greater than 1 for a given muscle action indicates that it is overloaded. Activity was determined for each muscle action in the model, with the total muscle activity of all the muscles being the sum of the activity of all muscle actions included in the model. The total muscle activity is the sum of the activity of all muscle actons building the model.

Muscle fatigue was expressed by the value of optimization task’s objective function, which should be interpreted as follows: the higher the function value, the higher the muscular fatigue. An adopted optimization criterion was the criterion of movement control assuming minimization of the cubic sum of the proportion of the muscular force to the maximum force.

#### 2.3.3. Models of Older Females Incorporating Posture and Strength Loss Associated with Natural Aging and Progressive Sarcopenia

Based on experimental studies, two variants of the posture of older females with different trunk and pelvic tilt angles in the sagittal plane (mean angles taken from experimental studies) and constant knee flexion (γ = 10°) reflect, respectively:

- The posture of OF as a result of the natural aging processes (M-NA).
- The posture of OFS as a result of progressive sarcopenia (M-SP).

The simulations assumed a percentage loss of maximum muscle strength corresponding to an age of 80 (the age for the modeling closest to the mean age in the OF and OFS study groups). Simulations were performed for the 50th percentile female model (65 kg and 1.65 m).

Statistical analyses were not performed on the deterministic model outputs, which represent single paired simulations at consecutive time points for two conditions (natural aging and sarcopenia). These results are not independent samples and therefore do not meet the assumptions of inferential statistical methods. The results presented in the figures serve solely to illustrate the modeled trends.

## 3. Results

This section presents experimental findings and results of modeling studies on age-related changes in the muscle system of older females, resulting from natural aging and processes associated with progressive sarcopenia.

### 3.1. Experimental Results

The DEXA-derived body composition data are presented in Table 1. The ALM determined by DEXA tests on the study group was 6.25 ± 0.66. ALM results were 5.07 ± 0.42 for OFS (10 subjects) and 7.44 ± 0.9 for OF. Average age for women in the OF group: 76 ± 4, in the OFS group: 80 ± 7. Compared to older females without sarcopenia (OF), participants with sarcopenia (OFS) showed lower muscle mass, while fat mass and body fat percentage were similar between the groups, reflecting distinct differences in muscle profiles. Despite similar levels of adiposity between the groups, participants with sarcopenia (OFS) exhibited significantly lower muscle mass. These findings underscore the presence of distinct muscular deficits in sarcopenic individuals, independent of overall adiposity, and are consistent with the phenotype of sarcopenic obesity.

Table 2 shows the trunk tilt (α) and pelvic tilt (β) values in the sagittal plane for the OF and OFS groups.

### 3.2. Modeling Changes in the Muscle System of Older Females

**Figure 3:**
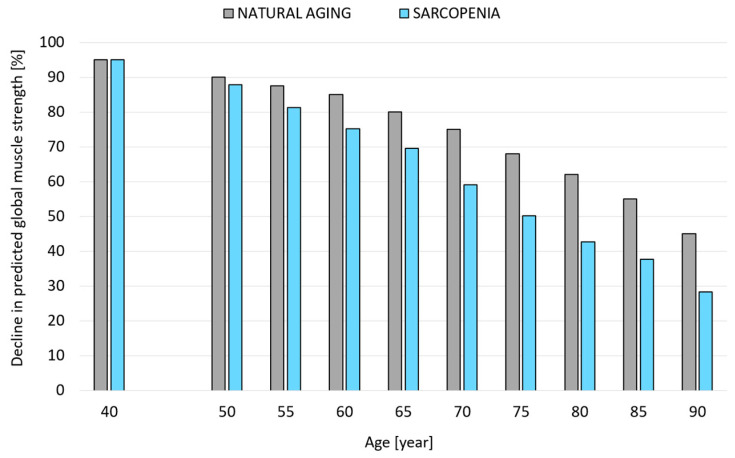
A model of changes in muscular strength in older females that accounts for the percentage loss of maximum muscular strength with age in older people due to natural aging processes and progressive sarcopenia.

Figure 3 shows a model of changes in the muscle system of older females that accounts for the percentage loss of maximum muscle strength with age in older people due to natural aging processes and progressive sarcopenia.

### 3.3. Model Results

**Figure 4:**
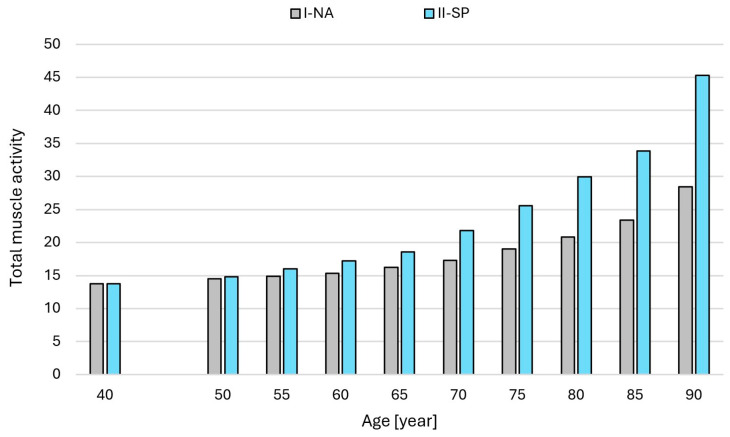
Total muscle activity for the two simulation variations (I-NA and II-SP). Legend: I-NA—simulation variant accounting for the aging process defined by a model that assumes a percentage loss of maximum muscular strength with age due to the natural aging process; II-SP—simulation variant accounting for the aging process defined by a model assuming a percentage loss of maximal muscle strength with age in individuals with progressive sarcopenia; Total muscle activity—the dimensionless sum of the activity of all muscle actions building the model. Note: Statistical analyses were not performed, as the data represent deterministic model outputs at consecutive time points for paired conditions (natural aging and sarcopenia). The results illustrate modeled trends rather than independent samples suitable for inferential statistics.

**Figure 5:**
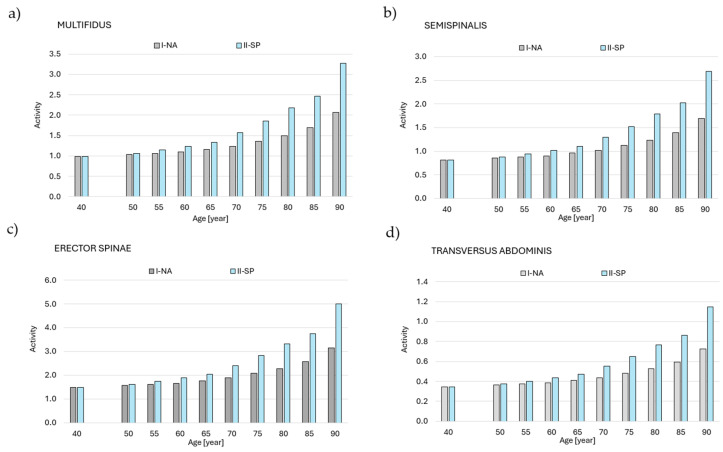
Summary muscle activity value for (**a**) multifidus, (**b**) erector spinae, (**c**) semispinalis, and (**d**) transversus abdominis for I-NA and II-SP simulations. Legend: I-NA—simulation variant accounting for the aging process defined by a model that assumes a percentage loss of maximum muscular strength with age due to the natural aging process; II-SP—simulation variant accounting for the aging process defined by a model assuming a percentage loss of maximal muscle strength with age in individuals with progressive sarcopenia; MULTIFIDUS—Multifidus muscle; SEMISPINALIS—Semispinalis muscle; ERECTOR SPINAE—Erector spinae muscle; TRANSVERSUS ABDOMINIS—Transversus abdominis muscle; Activity—is a dimensionless ratio of muscle force to its maximum force (0–1), with values >1 indicating overload. Note: Statistical analyses were not performed, as the data represent deterministic model outputs at consecutive time points for paired conditions (natural aging and sarcopenia). The results illustrate modeled trends rather than independent samples suitable for inferential statistics.

**Figure 6:**
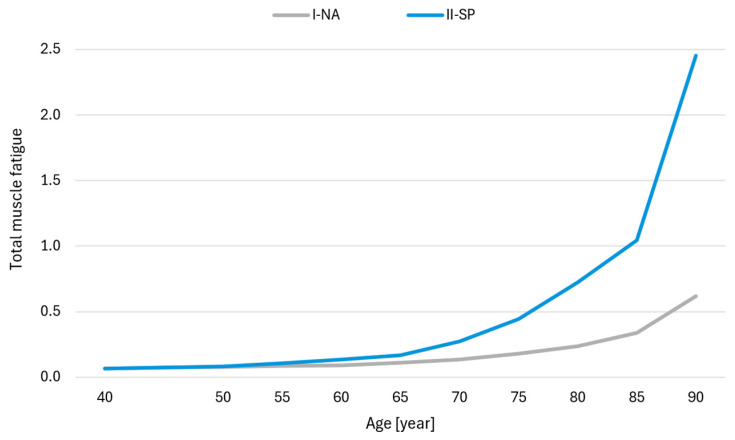
Muscle fatigue for I-NA and I-SP simulations. Legend: I-NA—simulation variant accounting for the aging process defined by a model that assumes a percentage loss of maximum muscular strength with age due to the natural aging process; II-SP—simulation variant accounting for the aging process defined by a model assuming a percentage loss of maximal muscle strength with age in individuals with progressive sarcopenia; Total muscle fatigue—a dimensionless indicator from the optimization function, where higher values reflect greater fatigue. Note: Statistical analyses were not performed, as the data represent deterministic model outputs at consecutive time points for paired conditions (natural aging and sarcopenia). The results illustrate modeled trends rather than independent samples suitable for inferential statistics.

Figure 4 shows the total muscle activity of all muscles included in the model for the two simulation variations (I-NA and II-SP) in subsequent years of the lifespan. Based on the analysis, the highest activity was observed in the following back and abdominal muscles (Figure 5): multifidus, ES, semispinalis, and TrA. Figure 6 shows a graph of muscle fatigue for the whole model in the two simulation variants in subsequent years.

### 3.4. Results of Models Accounting for Posture and Loss of Strength Capacity Associated with Natural Aging Processes and Progressive Sarcopenia

**Figure 7:**
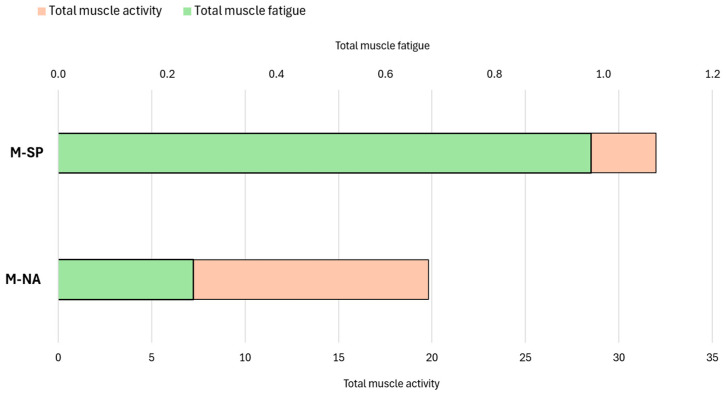
Total muscle activity and muscle fatigue for M-NA and M-SP models. Legend: M-NA—model of the posture of OF and strength loss associated with natural aging processes; M-SP—model the posture of OFS and strength loss associated with progressive sarcopenia; Total muscle activity—the dimensionless sum of the activity of all muscle actions building the model; Total muscle fatigue—a dimensionless indicator from the optimization function, where higher values reflect greater fatigue.

Figure 7 shows the total muscle activity of all muscles and muscle fatigue for two older females considering the posture and loss of strength associated with M-NA and M-SP.

## 4. Discussion

This study analyzed the effects of age-related muscle strength loss and sarcopenia on muscle function during standing in older females by studying posture and modeling changes in muscle strength with age due to natural aging and progressive sarcopenia. Model studies evaluated changes in total muscle activity and fatigue, loss of strength capacity and posture changes due to age-related muscle strength loss, and sarcopenia.

The model results support the hypothesis that aging is associated with an increase in total muscle activity while maintaining a standing posture and that sarcopenia leads to a dynamic increase in this activity, particularly after the age of 65. The results also confirmed the hypothesis that there is a marked increase in muscle fatigue while maintaining a standing posture after 65 years of age in those with progressive sarcopenia.

Muscle activity gradually changes in older people due to natural aging, with associated changes in muscle structure and function. With age, the muscles become weaker, and their mass and ability to generate force decrease [5,17,18,19,20,41]. These changes lead to increased muscle activity, which affects daily functioning and quality of life [42].

The results showed that simulated muscle aging significantly affected muscle function, particularly in the context of progressive sarcopenia. In the I-NA model, total muscle activity remained relatively stable until around 65 years of age, after which it increased moderately, exceeding 20 by age 80. In contrast, in the II-SP model, activity began to rise earlier, from the age of 50, and by 80 years it reached nearly 30, more than 40% higher than in the I-NA model (Figure 4). This accelerated increase reflects the compensatory recruitment of additional muscle groups to counter sarcopenia-related weakness, leading to substantially higher postural loading in later life [20].

These observations align with the model study of Ignasiak et al. [25] which showed that natural muscle aging had little effect on muscle activity, while more severe stages of sarcopenia significantly increased muscle activity, with the back extensors, semispinalis, and abdominal muscles showing the highest activity levels. In the advanced stages of sarcopenia, these muscles work to the limit of their capacity, leading to an increased risk of fatigue and spinal instability.

Postural muscles are essential for maintaining balance and stability. Age-related strength decline, especially in sarcopenia, compromises their function, increasing the risk of falls [8,9,10]. Older people with sarcopenia often find it difficult to stand, walk, or get up from a chair due to weakened postural muscles, which are responsible for body stability, making them unable to counteract gravity effectively and leading to a vicious cycle in which reduced exercise speeds up muscle mass loss, limiting the ability to exercise [20].

Based on the analysis, the highest activity was observed in the following muscles of the back and abdomen (Figure 5a–c): multifidus, ES, semispinalis, and TrA. The curve for the increase in total muscle activity (Figure 4) was not significantly different from the sum of the muscle activity value curve at each age interval. As in the case of the total activity of all the muscles, the I-NA variant was generally characterized by lower muscle activity values and a more uniform increase in this activity in the following years of life compared to the II-SP variant. Up to the age of 55, the sum of the muscle activity was similar between the I-NA and II-SP variants. From age 60, there was an increase in the activity of individual muscles, with the difference between the simulation variants increasing from year to year. The highest muscle activity values were recorded for ES (Figure 5d). The results also suggest that changing posture in the elderly and those with sarcopenia also affects musculoskeletal function (Figure 7).

Muscle fatigue increases with age (Figure 6), as shown by the simulation results. The initial exhaustion levels were relatively low in the I-NA variant, though they increased by 9% over five years between ages 50 and 60. The rate of increase in muscle fatigue accelerated after 60, with fatigue increasing by approximately 20% in five years between ages 60 and 70. From 70 to 80, fatigue increased by more than 30% and over 40% in the following five years (up to age 85). Muscle fatigue was higher for the II-SP variant than for the I-NA variant. The rate at which fatigue increased between 50 and 65 remained relatively constant at 26–27% over five years, but there was a marked increase in muscle fatigue after 65. Between 70 and 80, fatigue went up by 62–65% over five years, and by age 80, it was more than three times higher in II-SP than in I-NA. The following five years (up to 85) showed a 45% increase, while muscle fatigue was almost four times higher for the II-SP than the I-NA at 90.

These results reflect the known physiological basis of sarcopenia, including selective loss of fatigue-resistant fibers and reduced neuromuscular efficiency [4,42]. These impairments increase fatigue even at low activity levels, contributing to frailty and impaired mobility.

Physical inactivity is a major secondary factor that contributes to muscle aging [43,44,45]. In older people and those with sarcopenia, it is vital to implement exercise programs that include postural and strength muscles to slow sarcopenia progression and improve muscle activity and quality of life. Research suggests that regular exercise can also improve muscle perfusion, promote recovery, and delay the onset of fatigue [26,46]. The preservation of lean mass and prevention of fat gain may also be critical for maintaining strength and muscle quality in older age [26]. Strengthening the deep abdominal and back muscles can be particularly helpful for older people. This type of exercise helps relieve spinal load [36,43,44], improves posture, balance, and can reduce the risk of falls.

### Limitations to This Work and Directions for Further Research

An important limitation of this study stems from modeling muscle strength loss based solely on the reduction of PCSA. The strength capacity of individual muscles is also influenced by factors such as muscle composition [47], metabolism and neural activation [48], muscle fiber type and angle [49]. The loss of strength in later life is not equal between the upper and lower limbs, with greater reductions in strength generally found in the lower limbs [50]. A further limitation is the use of a 50th percentile model, which allowed us to isolate the effects of muscle strength decline and postural changes, but did not account for individual variability in mass distribution or joint properties.

The next step for our modeling studies may be to examine the unequal decrease in muscle strength performance on muscle function in those with sarcopenia. Further research could focus on analyzing how postural changes with age, including sarcopenia, affect muscle function and musculoskeletal load. Future research should apply multivariate modeling to explore how unequal strength loss and postural adaptations affect musculoskeletal load in people with sarcopenia.

## 5. Conclusions

In conclusion, aging is associated with increased total muscle activity while standing, while sarcopenia leads to a faster increase, especially after the age of 65. At 65, total muscle activity was 15% higher in the OFS model than in the OF model, while the difference was 44% at 80. After age 65, muscle fatigue increased considerably in females with progressive sarcopenia. Model studies have shown that, at age 80, muscle fatigue during standing (only due to changes in muscle strength) in females with sarcopenia can be more than three times higher than in females of the same age without sarcopenia (subject only to natural aging processes).

## Figures and Tables

**Table 1:** Anthropometric and DEXA-derived body composition characteristics of older females without (OF) and with sarcopenia (OFS).

Parameters	OF (*n* = 10)	OFS (*n* = 10)
Age (years)	76 ± 4	80 ± 7
Height (m)	1.57 ± 0.06	1.57 ± 0.05
Weight (kg)	65 ± 12	70 ± 7
BMI (kg/m^2^)	26.5 ± 5.3	28.2 ± 3.4
ALM (kg/m^2^)	6.25 ± 0.42	5.07 ± 0.42
Muscle mass (%)	41.1 ± 3.2	35.1 ± 4.1
Body fat (%)	40.3 ± 7.2	41.8± 3.6

Legend: Values are presented as mean ± standard deviation. OF—older females without sarcopenia; OFS—older females with sarcopenia; BMI—body mass index; ALM—appendicular lean mass index; Muscle mass (%)—percentage of total skeletal muscle mass; Body fat (%)—percentage of total body fat.

**Table 2:** Results of postural tests (trunk tilted and pelvis aligned in the sagittal plane).

Parameters	OF	OFS	*p*-Value (Cohen’s d)
Trunk tilted in the sagittal plane (α) [°]	5.1° ± 3.8°	12.2° ± 6.2°	0.008 * (−1.38)
Pelvis aligned in the sagittal plane (β) [°]	9.7° ± 5.3°	14.3° ± 7.3°	0.126 (−0.72)

Legend: Values are presented as mean ± standard deviation. OF—older females without sarcopenia; OFS—older females with sarcopenia; * indicates statistical significance (*p* < 0.05).

## Data Availability

The raw data supporting the conclusions of this article will be made available by the authors on request.
